# A novel synthetic DNA vaccine elicits protective immune responses against Powassan virus

**DOI:** 10.1371/journal.pntd.0008788

**Published:** 2020-10-29

**Authors:** Hyeree Choi, Sagar B. Kudchodkar, Michelle Ho, Emma L. Reuschel, Erin Reynolds, Ziyang Xu, Devivasha Bordoloi, Kenneth E. Ugen, Pablo Tebas, Joseph Kim, Mohamed Abdel-Mohsen, Saravanan Thangamani, David B. Weiner, Kar Muthumani

**Affiliations:** 1 Vaccine & Immunotherapy Center, The Wistar Institute, Philadelphia, Pennsylvania, United States of America; 2 Department of Microbiology and Immunology, SUNY Center for Environmental Health and Medicine, SUNY Upstate Medical University, Syracuse, New York, United States of America; 3 Department of Molecular Medicine, University of South Florida Morsani College of Medicine, Tampa, Florida, United States of America; 4 Division of Infectious Diseases, Perelman School of Medicine, University of Pennsylvania, Philadelphia, Pennsylvania, United States of America; 5 R&D, Inovio Pharmaceuticals, Plymouth Meeting, Pennsylvania, United States of America; University of Texas Medical Branch, UNITED STATES

## Abstract

Powassan virus (POWV) infection is a tick-borne emerging infectious disease in the United States and North America. Like Zika virus, POWV is a member of the family Flaviviridae. POWV causes severe neurological sequalae, meningitis, encephalitis, and can cause death. Although the risk of human POWV infection is low, its incidence in the U.S. in the past 16 years has increased over 300%, urging immediate attention. Despite the disease severity and its growing potential for threatening larger populations, currently there are no licensed vaccines which provide protection against POWV. We developed a novel synthetic DNA vaccine termed POWV-SEV by focusing on the conserved portions of POWV pre-membrane and envelope (prMEnv) genes. A single immunization of POWV-SEV elicited broad T and B cell immunity in mice with minimal cross-reactivity against other flaviviruses. Antibody epitope mapping demonstrated a similarity between POWV-SEV-induced immune responses and those elicited naturally in POWV-infected patients. Finally, POWV-SEV induced immunity provided protection against POWV disease in lethal challenge experiments.

## Author summary

Powassan virus (POWV) is an emerging RNA virus, belonging to the tick-borne flavivirus family and transmitted to humans through the bite of an infected tick. Infection can produce severe neurological manifestations, including meningitis and encephalitis, leading to death. Despite the potential for its emergence, currently antiviral therapies are not available to treat or prevent this emerging infection. This situation calls for concern and needs to be addressed. In this study, we have designed and produced a consensus, synthetic enhanced vaccine (SEV) against POWV (POWV-SEV) that focuses on regions of the envelope protein. The effectiveness of this vaccine was evaluated murine models. We have tested the antigen-specific humoral responses to the POWV-SEVs including the induction of neutralizing antibody responses. In addition, cellular immunogenicity including identification of dominant epitopes and polyfunctionality of cytokine-producing T-cells were characterized in POWV-SEV administered mice. Finally, we assessed the protective efficacy of POWV-SEV using a murine challenge natural infection model of POWV. These studies are highly novel and support the feasibility of developing an envelope-based synthetic enhanced DNA vaccine to aid in mitigating the public health threat emerging tick-borne viruses may pose to outdoor pets and humans in endemic areas.

## Introduction

Powassan virus (POWV) is a tick-borne member of the family Flaviviridae, first reported in 1958 [1–4]. It is the only tick-borne member of the genus *Flavivirus* with human pathogenicity in North America. Small and medium-sized mammals are common reservoirs notably, woodchucks and white-footed mice, and several species of tick act as vectors [1, 5]. Notably, this virus is the only known agent causing tick-borne encephalitis in North America. It is divided into two lineages: lineage I is called Powassan virus, whereas lineage II is known as deer-tick virus (DTV) [6, 7]. These two genetic lineages are distinguished by a 15% difference in the nucleotide sequences and a 2.9% difference in amino acid sequence in envelope (E) protein while the nonstructural region constitutes an 11.1% difference in nucleotides and a 5.4% difference in amino acids. The genetic variations between POWV and DTV so far do not warrant separate species as the variations are within similar parameters of other flaviviruses [8]. The POWV lineage is transmitted by a variety of tick species including but not limited to *I*. *cookei*, *Ixodes marxi*, *T*. *hudsonicus*, *Marmota monax*, and *Mephilis sp*., whereas the DTV lineage is primarily transmitted by *I*. *scapularis*, whose circulating range has been rapidly expanding due to warming climates [1, 5].

Clinically, both genetic lineages cause meningoencephalitis in humans, and infections with either lineage is serologically and symptomatically indistinguishable [5, 9, 10]. Pathogenesis is due to lymphocytic infiltration of perivascular neuronal tissue with a predilection for gray matter, including thalamus, midbrain, and cerebellum [11]. Patients with POWV infection typically exhibit encephalitis after an incubation period of 1–4 weeks [11, 12]. Approximately 10% of the reported infections have been fatal, and an additional 50% have produced long-term neurologic sequelae, including hemiplegia and headaches. The disease typically induces different symptoms like fever, body aches, skin rash and is potentially fatal to infected persons [13, 14]. Cases of POWV infections in human have risen ten-fold from the 1990s to 2010s, increasing from an estimated 1 case per year to 10 cases per year [5]. Therefore, it is highly imperative to consider an overall lack of aggregated data, that there is likely under-reporting of actual infections, as well as likely selective reporting of POWV neuroinvasive disease cases to the Centers for Disease Control and Prevention (CDC) when describing the possible growth in importance of POWV disease. The expansion of the POWV-carrying vector population as well as the dramatic increase in circulating infections in the U.S. indicate an endemic potential with possible severe clinical consequences for the North American residents [15–17].

As of 2019, there is only one POWV-specific vaccine in development, reported by VanBlargan et al [18]. This LNP-mRNA-based POWV vaccine induces potently neutralizing antibodies in a murine model, conferring full protection against both lineages of POWV as well as distantly-related Langat virus (LGTV) (*flaviviridae*: *flavivirus*) [4, 19]. Further, the POWV mRNA vaccine cross-protects against LGTV in a non-complete, yet statistically significant manner in terms of viral loads determined by qRT-PCR in blood, spleen, brain, and spinal cord (data not shown for POWV lineage I and II challenges) [19, 20].

Here we describe the use of a synthetic DNA-based vaccine platform in developing a vaccine against POWV that elicits both humoral and cellular immunity as previously reported for several other viral vaccine targets which have already been advanced to the clinic [21, 22]. To design this vaccine, a POWV-specific antigen cassette was designed that encodes a synthetically aligned consensus sequence spanning the prM and envelope portions for both POWV lineage I and II. This synthetic antigen was cloned into a pMV101 vector to create the POWV-SEV DNA vaccine. Electroporation-enhanced intramuscular immunization of animals with POWV-SEV induced potent POWV-specific cellular and humoral responses in C57BL/6 mice, which allowed us to test the efficacy of the vaccine *in vivo*. Immunization with the POWV-SEV DNA vaccine conferred complete protection in C57BL/6 mice from a lethal POWV challenge with no detectable viral load in various organ tissues and brain compared to the vehicle-vaccinated group. The significant protection offered in animals by this POWV-SEV DNA vaccine is highly encouraging and strongly supports the efficacy of this important platform at low doses of immunogens. Hence, extensive research to test this POWV-SEV DNA vaccine for human and veterinary protection is certainly warranted.

## Results

### Generation and characterization of synthetic enhanced vaccine (SEV) against POWV

Synthetic DNA (sDNA) vaccines have been studied in multiple clinical trials for different infectious diseases [21–26]. sDNA is attractive for several reasons including a well established safety profile, simple and rapid engineering and production with a clear path for clinical development. In addition, anti-vector immunity is not an issue as described for several other viral vaccines [27]. Further, sDNA vaccines are non-alive, non-replicating, and non-spreading, which limits viral reversion concerns reported to arise in case of some vectors [28]. A major effective approach for preventing viral infection is likely by inhibiting the entry of the virus particles into the target cells. Accordingly, we designed a synthetic consensus immunogen focusing on the viral envelope of POWV that induces both cellular and humoral immune responses with high durability and protects animals from POWV infection.

**Fig 1:**
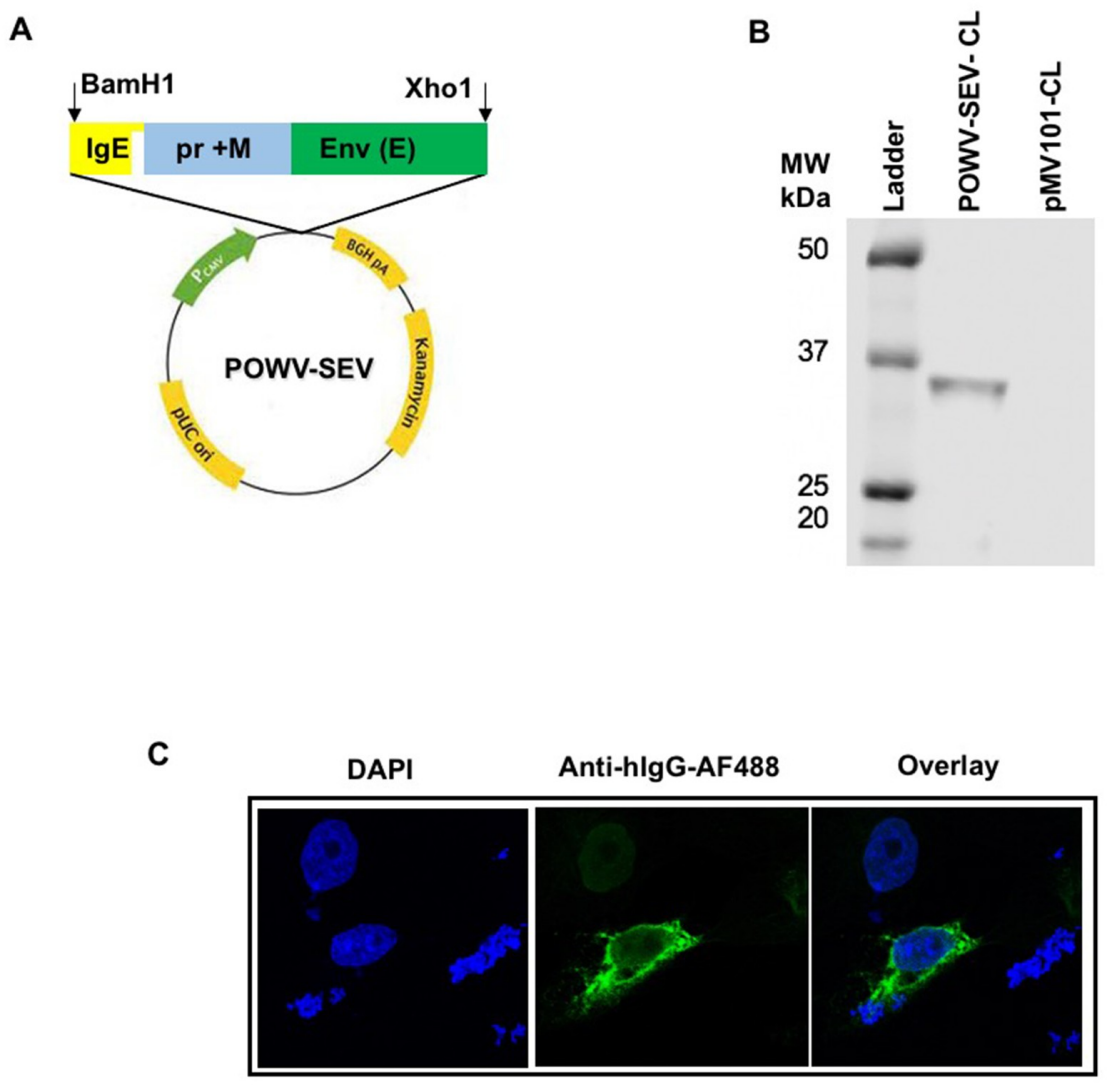
Development of POWV-SEV vaccine. (**A**) A schematic representation of the POWV-SEV construct. (**B**) POWV antigenic protein expressions by SDS-PAGE using Western blot analyses. POWV-SEV vaccine construct- or pMV101(empty vector) -transfected 293T cell lysates (CL) were incubated with anti-Flavivirus group antigen antibody (1:500). (**C**) Immunofluorescence Analysis (IFA) of POWV-SEV expression *in vitro*. IFA analysis for POWV-SEV protein expression in vero CCL-81 cells. Fourty-eight hours after transfection, immunofluorescence labeling was performed by incubating transfected cells with the anti-Flavivirus group antigen antibody (1:500) followed by the addition of goat anti-mouse IgG-AF 488 antibody (1:4000) for fluorescence conjugation. Nuclear DNA is stained with DAPI.

An overall consensus synthetic enhanced POWV-envelope sequence was developed based on an analysis of the alignment of current POWV pre membrane (prM) and envelope protein sequences (Fig 1A). This synthetic vaccine is termed as POWV-Synthetic Enhanced DNA Vaccine (POWV-SEV). POWV-SEV was placed in a modified pMV101 mammalian vector under the control of the CMV promoter in frame with a highly efficient IgE leader sequence, which was used to target recombinant proteins into endoplasmic reticulum to facilitate antigen expression [15, 26, 29]. The expression of this consensus antigen of POWV-SEV construct was demonstrated by Western blot using POWV-SEV-transfected 293T cell lysates collected at 48 hours probed with anti-Flavivirus group antigen antibody (Fig 1B). Immunofluorescence assay (IFA) analysis using POWV-SEV-transfected vero CCL-81 cell probed with commercially available anti-Flavivirus group antigen antibody, 48 hours post transfection, verified the expression of this contruct *in vitro*.

### Antibody responses after vaccination with SEV-encoding POWV-envelope

**Fig 2:**
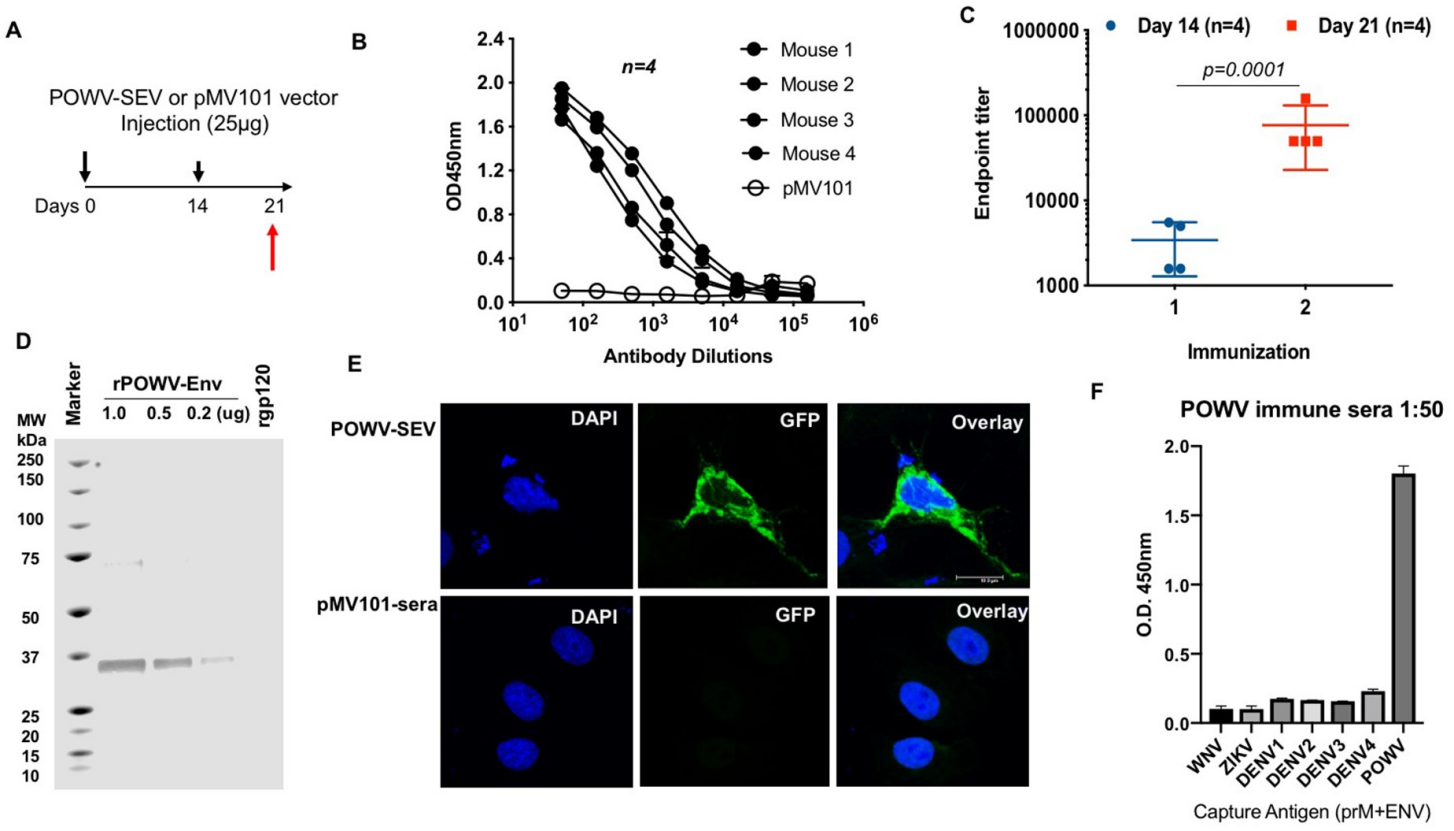
POWV-SEV vaccine induces functionally binding antibodies in mice. (**A**) A POWV-SEV-immunization schedule of C57BL/6 mice over 21 days. (B) Evaluation of antigen-specific antibody responses following immunization of mice with POWV-SEV vaccine at day 21. C57BL/6 mice were immunized two times at day 0 and 14, where 25μg of POWV-SEV DNA or pMV101 empty vector was delivered intramuscularly (i.m.) using EP-enhanced delivery system. Sera were collected one week after the 2^nd^ immunization at day 21. Half-log dilutions of day 21 immune sera from individual mice (*n = 4*) were evaluated for their binding capacity to a recombinant POWV-envelope protein at 1μg/ml concentration. The binding capacity of day 21 sera from pMV101-vaccinated mice were depicted as a single group called pMV101 (unfilled circles). (**C**) The endpoint titer of POWV-SEV-specific IgG post one and two immunizations. The antibody endpoint titer was defined as the highest dilution of a serum sample with OD values > (mean ±SD; *n = 4*) of vector vaccinated mice. Samples with a titer <50 were given an endpoint titer of 1. (**D**) Western blot analysis of POWV-SEV immunized murine sera. Pooled day 21 immune sera from the aforementioned experiment was used as a primary antibody to probe POWV-envelope protein (rPOWV-ENV in μg). An irrelevant recombinant gp120 protein (1.0μg) was used as a negative control. (**E**) Indirect immunofluorescence assay of POWV-SEV transfected Vero CCL-81 cells probed with POWV-SEV sera. POWV-SEV immune sera (day 21) obtained from vaccinated mice and pMV101-sera obtained from the vehicle-vaccinated group were diluted at 1:50 and placed on to slide chambers with POWV-SEV-transfected Vero CCL-81 cells. Further probing with goat anti-mouse IgG-AF-488 illuminates areas where POWV-envelop antigens are identified by POWV-SEV murine sera. DAPI nuclear staining is shown as blue. (**F**) Non-cross-reactivity of POWV-SEV immune sera against flavivirus envelope antigens-WNV, ZIKV, DENV 1–4, POWV. Day 21 POWV-SEV murine immune sera (1:50) is probed against flavivirus envelope antigens.

Following the expression assessment of POWV-SEV vaccine *in vitro*, we assessed immunogenicity induced by POWV-SEV immunization in C57BL/6 mice. Cohorts of mice (*n = 4*) were vaccinated 2 times (day 0 and day 14) with either 25μg of POWV-SEV or the vector backbone as a vehicle (pMV101), intramuscularly followed by 3P CELLECTRA electroporation (Fig 2A). Blood was collected at day 0, day 14 and day 21. To evaluate serum antibody responses, sera harvested at one week after the second vaccination (day 21) were subject to ELISA against plates coated with recombinant POWV-envelope protein at 1μg/mL. Mice immunized with 25μg of POWV-SEV developed high levels of detectable antibodies (Fig 2B), while no antigen-specific antibodies were detected in the pMV101 vehicle-vaccinated cohort. As shown in Fig 2C, the POWV-SEV immunized mice seroconverted after a single immunization where sera were collected day 14. Immune sera collected after a second immunization (day 21) exhibited a notable increase in the endpoint titer since the first immunization. Day 21 immune sera were also evaluated by Western blot analysis using varying amount of recombinant POWV-envelope protein, and an irrelevant recombinant protein (gp 120) as a loading control (Fig 2D). Probing with day 21 murine sera exhibited a specific reactivity to recombinant POWV envelope protein, found at 35 kDa, as expected. We also evaluated the serum reactivity by IFA using POWV-SEV transfected Vero cells with vaccinated immune sera (day 21), which identified cytoplasmic and likely surface expression of POWV- envelope antigen only when probed with POWV-SEV day 21 immune sera and not with pMV101-immunesera (Fig 2E).

Furthermore, within the envelope protein, one of the three structural proteins of the flavivirus virion contains the important common antigenic determinants [5, 14, 30],. We evaluated the potential cross-reactivity of anti-flavivirus antibodies induced by POWV-SEV immune sera with other flavivirus envelope protein. POWV-SEV immunized antisera at day 21 were tested against envelope antigens of POWV, Zika, WNV, and dengue serotypes 1 through 4 by ELISA for binding specificity (Fig 2F). None of the other flavivirus-immunogens in this group yielded positive POWV-IgG ELISA test results for binding specificity. The humoral response induced by POWV-SEV were robust and reacted specifically with POWV antigen.

### POWV-SEV-induced immune sera reactivity as compared to POWV convalescent patient serum- Antibody specificity

**Fig 3:**
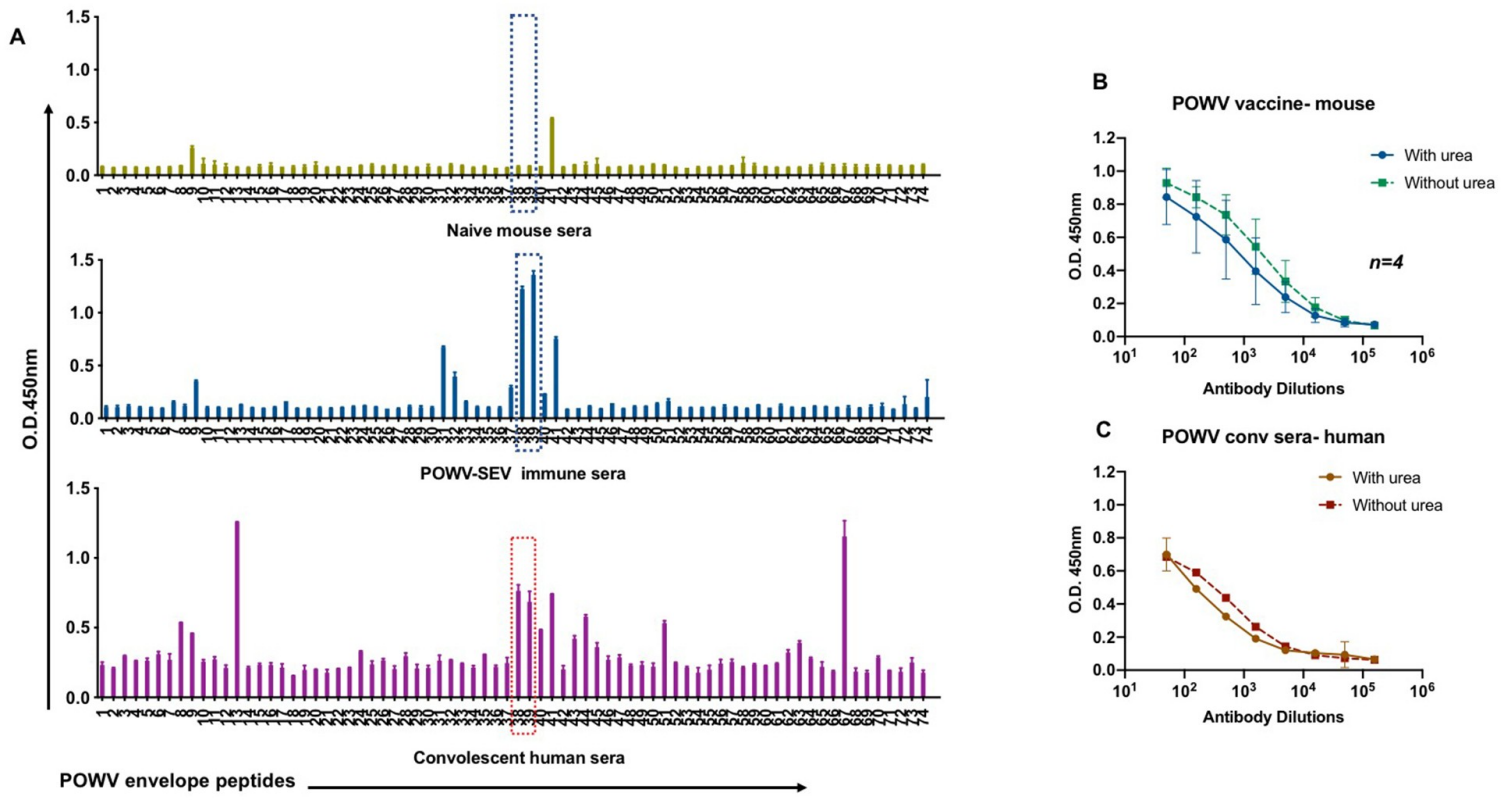
POWV-SEV -induced immune sera are comparable to POWV convalescent patient serum. Detection of IgG antibodies and IgG avidity in POWV- convalescent patient sera and POWV-SEV immunized sera. (**A**) Antibody mapping of sera from POWV- patients with a recent primary infection POWV with POWV-SEV envelop peptides spanning the entire length of the protein were evaluated specific IgG antibodies detected by ELISA. POWV-SEV pooled murine immune sera (day 21), pMV101 vector control-immunized sera and POWV convalescent patient sera were probed over 74 individual POWV envelope peptides (15-mers with 9 overlapping amino acid sequences). Assessment performed in duplicate. (**B**-**C**) IgG avidity in POWV-SEV immunized sera *(n = 4)* avidity compared to POWV convalescent sera. Antibody responses were assessed by ELISA.

The envelope proteins have a degree of structural similarity that may contribute to shared antibody epitopes. Therefore, it is important to compare immune reactivity from POWV-SEV vaccinated mice with that of sera from humans with documented prior exposures to POWV. We investigated the epitopes, binding avidities, and neutralization potencies of POWV-SEV vaccine induced immune responses. We tested serum samples from POWV-infected individuals to measure levels of binding antibodies activity and showed antibodies titers to recombinant POWV-Envelope protein. We next evaluated serum samples collected from convalescent POWV patients identified in Illinois to compare natural and vaccine-induced antibody responses (Fig 3A). ELISA binding data demonstrated high titers of POWV-envelope specific antibody in these patient sera, with some similarities to the antibodies in the sera of immunized animals. For example, the highest common responses in antibody in both natural infection and vaccine-generated immune sera were identified as peptide 38 and 39 after subtracting the background, denoting the POWV envelope region ‘ATLPEEHQA’. This POWV envelope region matched the B cell dominant epitope prediction algorithm blasted on IEBD Analysis Resource.

Furthermore, the relative avidity of POWV-Envelope specific IgG antibodies was determined by a urea ELISA. Antibody avidity was studied by treating serum with 4M Urea in the ELISA assay (Fig 3B and 3C). Among the POWV-IgG-positive convalescent samples, no significant difference in resistance to Urea treatment was observed between them and immune sera from POWV-SEV vaccinated animals. Convalescent patient sample exhibited high avidity indices for IgG1 antibodies indicating a higher avidity for both immune sera samples.

### POWV-SEV DNA vaccine elicits antigen-specific T cell responses in mice

**Fig 4:**
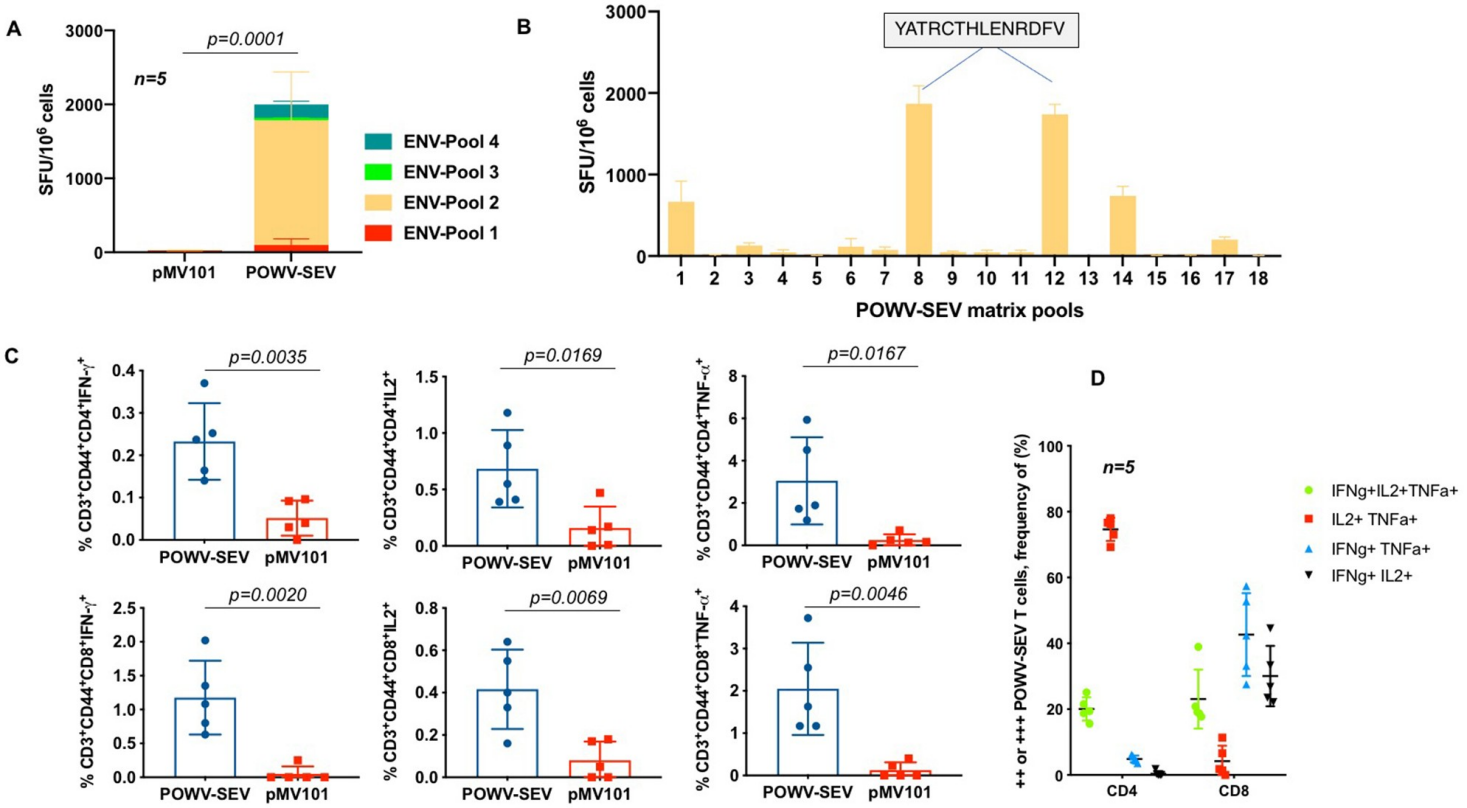
POWV-SEV vaccine elicits antigen-specific CD4^+^ and CD8^+^ T cell responses in mice. (**A-B**) ELISpot analysis of splenocytes secreting IFN-γ in response to POWV-SEV immunizations measured in spot-forming units (SFUs) per 10^6^ splenocytes. Harvested splenocytes of POWV-SEV vaccinated C57BL/6 mice (*n = 5*) were stimulated *ex vivo* with POWV envelope peptides spanning the entire length of the protein. (**A**) IFN-γ-secreting SFU per 10^6^ stimulated with linear peptide pools or (**B**) matrix peptide pools. The immunodominant epitope matched with the H2-Db-restricted prediction of dominant epitope is highlighted in arrows. (**C**) Percent intracellular cytokine population for antigen-specific CD4^+^ and CD8^+^ T cells. Splenocytes that were stimulated with POWV-envelope peptides spanning the entire length of the protein were evaluated for CD4^+^ and CD8^+^ T cells producing IFN-γ, IL-2, and TNF-α via flow cytometry. (**D**) Polyfunctionality of antigen-specific CD4^+^ and CD8^+^ T cells. Frequency of total CD4^+^ and CD8^+^ T cells expressing double- or triple- positive cytokines (IFN-γ, IL-2, and/or TNF-α) using Boolean gating strategy.

We have generated an immunogen focusing on the conserved portions of the POWV-envelope based on computer generated sequence analysis. Upon evaluation of humoral immune responses, we measured T cell responses to determine if the POWV-SEV vaccine could generate cellular immunity against envelope antigens in mice. Generation of antigen-specific T cells is critical in mediating immunopathology in vector-borne viral encephalitis [31, 32]. In order to evaluate T cell immune responses elicited by the POWV-SEV vaccination, we used the classical IFN-γ ELISpot assay on splenocytes harvested from mice following plasmid DNA immunization. The POWV-SEV-immunized mice possessed POWV-specific T cells against envelope antigens, as evidenced by an increase in the number of POWV peptide-induced INF-γ producing cells for pools 1–4 (Fig 4A).

Synthetic DNA vaccine mediated antigen expression proved successful in eliciting an increased number of Ag-specific CD8^+^ T cells in several studies. SEV vaccination was found to be highly T cell immunogenic. Since it is well-established that the diversity, or breadth, of the T cell response may be important for conferring protection, we aimed to evaluate the comprehensive epitopic T cell response as induced by POWV-SEV vaccine construct. We developed a modified IFN-γ ELISpot assay that identified and measured subdominant and immunodominant POWV-T cell epitopes. This was achieved by incubating samples with matrix peptide pools. The immunodominant epitope for POWV-SEV in mice with the C57BL/6 haplotype was subsequently determined to be the sequence “YATRCTHLENRDFV” and this comprising peptide (Fig 4B) was then confirmed by using the immune epitope database analysis resources tools and MHC-binding scores of the MHCpre software analysis (http://tools.iedb.org, http://www.syfpeithi.com/scripts/MHCSr.dll/home.htm; & http://tools.immuneepitope.org) followed by further characterization with the help of FACS analysis.

We next examined T cell responses from the spleen using several quantitative assays, including ICS focusing on the production of IFN-γ, TNF-α and IL-2. As shown in Fig 4C and 4D, POWV-SEV induced strong IFN-γ response against envelope antigens, as evidenced by the increase in the number of POWV peptide-induced IFN-γ, TNF-α and IL-2, producing CD4 and CD8 T cells. Thus, immunization with the POWV-SEV alone induces T-cell responses against envelope antigen. The results showed that the POWV-SEV immunized mice elicited T cell responses remarkably.

### Protective effects against POWV-challenge in mice immunized with POWV-SEV

**Fig 5:**
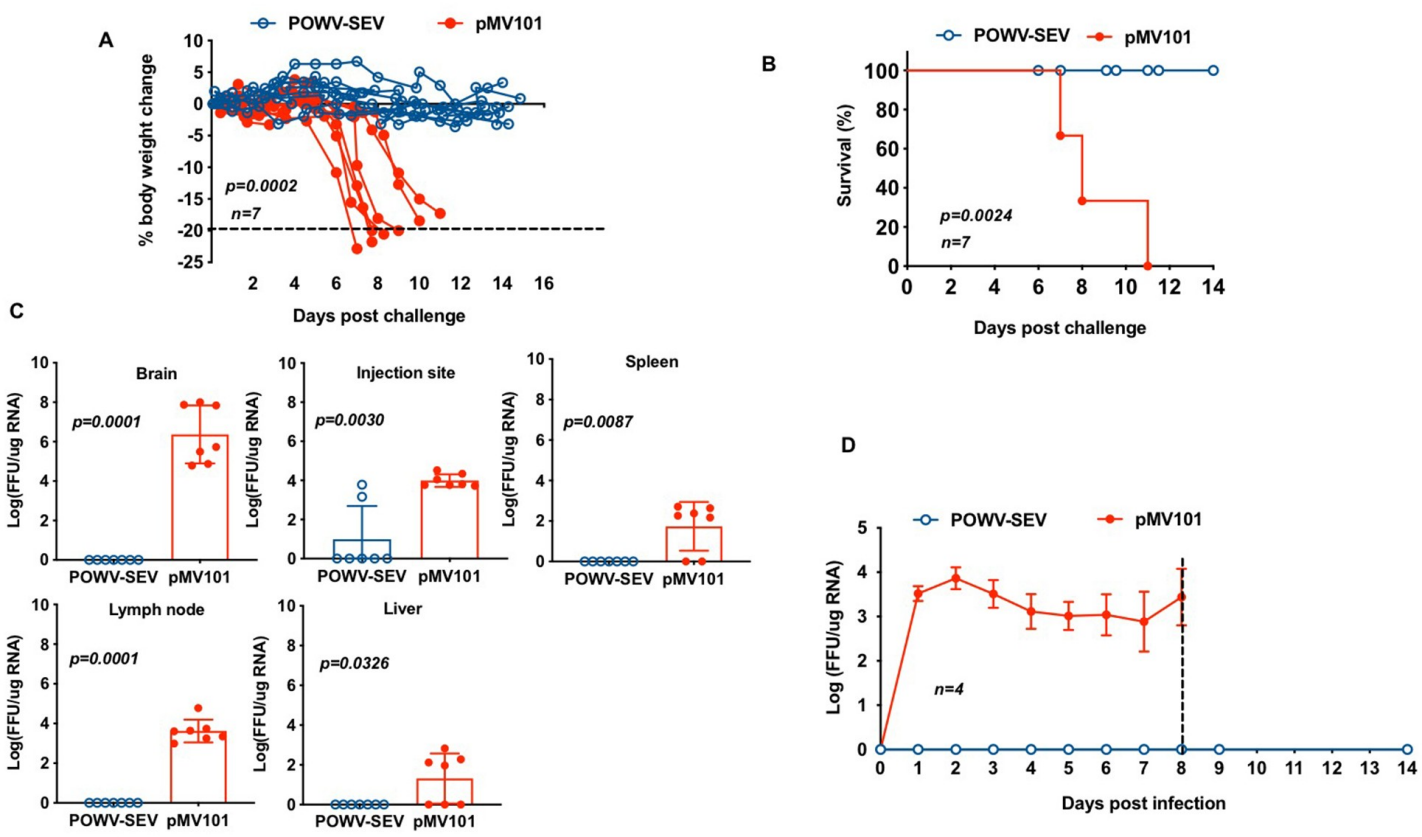
Immunizations with POWV-SEV vaccine confer protective efficacy in mice. C57BL/6 mice (seven per group) were immunized with POWV-SEV (blue circles) or mMV101 vector (red filled circles) and challenged with POWV virus. (**A**) The percent change from initial body weight, (**B**) Kaplan-Meier survival curve, (**C**) and organ viral loads (FFU/μg RNA) post POWV viral challenge are shown. (**C**) Powassan viral burden in organs of pMV101- or POWV-SEV vaccinated mice at endpoint or 14 days post infection, whichever came later. The average end point for POWV-challenged pMV101-control group was 8 dpi. POWV-challenged POWV-SEV group were sacrificed at day 14 post infection. (**D**) Kinetics of circulating peripheral blood viral load of pMV101 or POWV-SEV-vaccinated C57BL/6 mice upon challenge (*n = 7*) depicting average FFU/μg RNA of each cohort. The dotted line indicates the average survival in days for control pMV101 vaccinated mice upon challenge. Error bars (**C**) and (**D**) depict the standard error of the mean. Results were compared by using Student’s *t* test.

Since the POWV-SEV elicited high and homogenous envelope-specific cellular and humoral immune responses, we further tested the vaccine in challenge experiments to evaluate if these POWV-SEV-induced immune responses could protect mice against inoculation of live virus. Two groups (POWV-SEV or pMV101) of mice containing 7 each were inoculated intramuscularly with 25μg of POWV-SEV or pMV101 expression vector on day 0 and 14. One week after the second immunization (day 21), mice were inoculated with 10^4^ FFU of Powassan virus using a foot pad injection. Animals were monitored for the subsequent 28 days for mortality and morbidity, mainly regarding the development of clinical signs and symptoms. Mice have been previously reported to succumb to POWV encephalitis within 8 days post infection [30]. POWV challenged mice in the pMV101 control group lost significant amounts of weight and started to show clinical signs of disease, i.e., ruffled fur and weight loss at 8 days post infection (dpi) and progressed to show signs of severe neurological disease, characterized by hind limb paralysis and showed clinical signs of disease. Mice in the pMV101-control group started to show clinical signs of disease, i.e., ruffled fur and weight loss at 6 days post infection (dpi) (Fig 5A). All the mice in the pMV101-control group progressed to show signs of severe neurological disease, characterized by hind limb paralysis and showed clinical signs of disease i.e., ruffled fur and weight loss. Mice showed weight lost by 8 dpi and were euthanized if lost more than 15% of its original body weight. The group of mice vaccinated with POWV-SEV maintained their original body weight and did not exhibit any signs of POWV-related symptoms. POWV-ENV vaccinated mice were protected against infection (Fig 5B).

In order to quantify the level and extent of infection in mice, we measured POWV RNA by qPCR. Blood was collected from mice at 1, 3, 5, 7, 8, 10, and 14 days post infection (dpi) for virus titration. After sacrificing mice at their end point or at day 14 post infection, whichever came later, skin from POWV-injected footpad, popliteal lymph node, liver, spleen, and brain were harvested. The POWV-RNA genome copy numbers in the brains of C57BL/6 mice were more varied, depending on severity and disease outcome (Fig 5C). As shown in Fig 5D, the average POWV-RNA genome copy number was 3.3 x 10^7^, FFU/ μg RNA in the pMV 101-control-vaccinated mice. In contrast, POWV-SEV vaccinated mice showed no detectable POWV-RNA in serum. Taken together, the data from this study demonstrate that mice immunized with a synthetic DNA vaccine encoding a POWV-envelope antigen induced protective immunity against Powassan viral infection.

## Discussion

The severity of POWV-induced pathology and the recent re-emergence of the disease as a result of increased population growth and habitat expansion of its tick vectors make developing a vaccine to POWV an urgent goal of the scientific community [5, 6, 12]. Currently, there is no specific vaccine or distinct antiviral treatment for POWV, which highlights the urgent need to develop a vaccine against this disease. Here, we developed a novel synthetic DNA vaccine for POWV that elicited protective immune responses and protected mice from POWV infection. DNA, as a vaccine platform, has been studied in numerous clinical protocols related to infectious diseases [23, 26, 29]. DNA is attractive because of its improved safety profile compared to live attenuated vaccines and also due to the simplicity of engineering and production compared to the development of live attenuated and killed viral vaccines. In addition, anti-vector immunity is not an issue [23]. In the initial studies performed over 20 years ago, DNA vaccines were found to produce extremely weak immune responses in non-human primate and in human clinical studies. Through substantial engineering of the plasmid vaccine insert, backbone modifications, formulation optimizations and use of effective electroporation technology, our group has increased DNA vaccine immunogenicity dramatically [15, 23, 29, 33]. These modifications have led to the induction of strong immune responses in the clinic including the first DNA vaccine to drive potent cell mediated and humoral immunity [21, 23], the first and only DNA vaccine to generate efficacy in a double blind placebo controlled clinical trial, and rapid progression from preclinical to the clinic for the first reported DNA vaccines for Zika [21], MERS-CoV [22] and SARS-CoV2 [34]. In theory, the synthetic DNA vaccine platform has a great deal to offer within the context of a DNA vaccine approach for POWV.

Our novel synthetic vaccine was subjected to combined enhancements of plasmid antigen design including codon optimization, RNA optimization, RNA stabilization and increased leader sequence utilization which are favorable over traditional vaccines owing to their safety, cost-effectiveness and ability to modulate immune responses against POWV to increase the chances of protection [21, 22, 24, 26, 29, 33]. Priming of mice with POWV-SEV was done by intramuscular injection followed by electroporation resulting in strong cellular and humoral immune responses measured by T cell ELISpot and ELISA assays. The novel synthetic DNA delivery substantially improved the resulting immune responses induced and this study suggests that such improved DNA constructs and delivery using EP may offer additional benefit.

Our synthetic vaccine is capable of eliciting both humoral and cellular immunity that are POWV envelope-specific. POWV-SEV vaccinated mice generated robust antigen-specific IgG with high avidity that are comparable to antibodies found in convalescent human sera. Moreover, the vaccine-induced IgG in mice and the POWV human sera matched the predicted B cell dominant epitope, suggesting that POWV-SEV was capable of generating immunogenicity similar to that generated by natural infection. POWV-SEV induced IgG in mice did not cross-react with other flavivirus antigens such as Zika, WNV, and all four serotypes of DENV as expected. Additionally, we demonstrated that POWV-SEV was capable of generating robust levels of antigen-specific CD4^+^ and CD8^+^ T cells producing IFN-γ, TNF-α, and IL-2. Evaluating antigen-specific CD8^+^ T cells are of particular importance given that CD8^+^ T cells mediate the inflammatory reaction pivotal in fatal tick-borne encephalitis [32]. We have previously demonstrated the immunogenicity and protection from murine viral challenges of several DNA vaccine candidates for emerging- and re-emerging infectious viruses, such as ZIKV, CHIKV, MERS-CoV, EBOV, MAYV and SARS-CoV2 [15, 21–23, 25, 26, 34]. Overall, POWV-SEV conferred 100% protection in POWV challenge in mice with no notable POWV viral loads detected in the vital organs except for the initial injection site.

In summary, we report a POWV-SEV vaccine using enhanced EP-delivery to be a viable platform for developing a vaccine against another flavivirus, Powassan virus. This highlights the utility of this platform for the development of vaccines against various vector-borne viral species and also points to the potential for optimizing adaptive immune responses so that they elicit specific immunity against circulating flaviviruses.

## Materials & methods

### Ethics statement

Animal experimentation was conducted following Wistar IACUC and Animal Facility guidelines for housing and care of laboratory animals and performed in accordance with recommendations in the Guide for the Care and Use of Laboratory Animals of National Institutes of Health (NIH). The Wistar Institutional Review Board (21610289) approved the studies involving human subjects.

### Cell culture

All primary cells and cell lines were cultured and incubated at 37°C and 5% CO_2_ setting. Human embryonic kidney 293T (HEK293T; ATCC-CLR-N268) and Vero CCL-81 (ATCC #CCL-81) (ATCC, USA) cells were cultured in D10 media using Dulbecco Modified Eagle’s Medium (Invitrogen Life Science Technologies, USA) supplemented with 10% heat-inactivated fetal bovine serum (FBS), 3 mM glutamine, 100 U/ml penicillin, and 100 U/ml streptomycin. Mouse splenocytes and cells in suspension were cultured in R10 media using RPMI1640 (Invitrogen Life Science Technologies, USA) medium supplemented with 10% heat-inactivated FBS, 3 mM glutamine, 100 U/ml penicillin, and 100 U/ml streptomycin [15].

### Virus propagation and titration for POWV challenge

Powassan Virus LB strain (POWV) was provided by the World Reference Center for Emerging Viruses and Arboviruses (WRCEVA, UTMB.) Virus propagation was completed in accordance with previously published methods [30]. Focus forming assay (FFA) to determine viral titer was adapted from previously published methods [37,38]. Briefly, Vero C1008 cells (E6; ATCC# CRL-1586) were grown on tissue culture (TC) -treated 12-well plates (Corning, USA), using Dulbecco’s Modified Eagle Medium (DMEM, Gibco, USA) supplemented with 10% Fetal Bovine Serum (Hyclone) and 1% Penicillin-Streptomycin (Corning, USA), at 37°C and 5% CO_2_. Following aspiration of media, serial dilutions of POWV were placed on cell monolayers for one hour. Wells from one column had media placed on them to serve as a negative control. Plates were gently rocked every 10 to 15 minutes for one hour to allow maximum contact with the cells. An overlay of 0.8% methylcellulose was added and plates were maintained for six days. Methylcellulose was removed and fixation was performed by adding methanol:acetone (1:1) for 30 minutes. Following fixation, the plates were allowed to air dry. Cell membrane permeabilization was performed by incubating with 0.5% Triton X-100 (Sigma-Aldrich, USA) in PBS and washing was done with 0.05% Tween20 (Sigma-Aldrich, USA) in PBS (PBST). A blocking solution of 5% goat serum (Sigma-Aldrich, USA) and 1% bovine serum albumin (Fisher, USA) in PBST was incubated on wells for one-hour followed by an additional hour incubation with mouse immune ascitic fluid against POWV (1:500) (World Reference Center for Emerging Viruses and Arboviruses (WRCEVA). Plates were then washed with PBST followed by one-hour incubation with goat anti-mouse IgG secondary antibody conjugated to horseradish peroxidase (1:1000) (Invitrogen, USA). Secondary antibody was washed and then AEC substrate was prepared and used in accordance with the ImmPACT AEC Peroxidase (HRP) Substrate kit (Vector Labs, USA) protocol, by adding in each well and allowing to develop in the dark.

### Construction of POWV-ENV DNA vaccine

The POWV-envelope plasmid DNA construct encodes full-length precursor of membrane (prM) and Envelope (E). A consensus strategy was used, and the consensus sequences were determined by the alignment of 20 current POWV prM + envelope protein sequences available in the Pubmed database. The vaccine insert was genetically optimized (i.e., codon and RNA optimization) for enhanced expression in human and an IgE leader sequence was added to facilitate expression [26, 34, 35]. The synthetic vaccine construct was designed and synthesized (GenScript, USA), then subcloned into a modified pMV101 expression vector under the control of the cytomegalovirus immediate-early promoter as described before [15]. The final construct is named POWV-SEV and the control plasmid backbone is pMV101 vector. Large-scale amplifications of DNA constructs were carried out and purified plasmid DNA was formulated in water for immunization. The sizes of the DNA inserts were confirmed via agarose gel electrophoresis. Phylogenetic analysis was performed by multiple alignment with ClustalW using MEGA version 5 software [15].

### Transfection, Western blot and immunofluorescence assays

HEK293T cells (ATCC-CLR-N268) were cultured using D10 media, and transfection was performed using the GeneJammer reagent, as per the manufacturer’s protocols (Agilent, Santa Clara, USA). Briefly, 3x10^5^ cells were seeded in a 12-well plate. After 24 hours transfection was performed with 1μg of POWV-SEV and GeneJammer (Agilent, USA). The cells were collected 48 hours post transfection, washed twice with PBS, and lysed with cell lysis buffer (Cell Signaling Technology, USA) and Halt Protease inhibitor cocktail (Thermo Fisher Scientific USA).

Western Blot analysis was done to verify the expression of the POWV-SEV. POWV-SEV transfected cell lysates and supernatants were run on precast 12% Bis-Tris NuPAGE gels (Life Technologies, USA) using the Odyssey protein Molecular Weight Marker (LI-COR Biosciences, USA) as a ladder. The gels were run at 200 V for 50 minutes in MOPS buffer. The proteins were transferred onto an Immobilon-FL PVDF membrane (EMD Millipore, USA) using the iBlot 2 Gel Transfer Device (Life Technologies, USA). The membrane was blocked in PBS Odyssey blocking buffer (LI-COR Biosciences, USA) for 1 hour at room temperature. To detect vaccine expression, the anti-Flavivirus group antigen antibody (EMD Millipore; Clone D1-4G2-4-15, USA) was diluted 1:500 in Odyssey blocking buffer with 0.2% Tween 20 (Bio- Rad). The membrane was incubated with primary antibody for overnight at 4 °C and then washed with PBST. The presence of POWV envelope protein was identified using goat anti-mouse IRDye680CW (LI-COR Biosciences, USA) for both MAB10216 and mouse sera. After washing, the membrane was imaged on the Odyssey infrared imager (LI-COR Biosciences, USA).

Western blot analysis to verify the presence of POWV-ENV IgG in vaccinated murine sera was performed in a similar manner. Varying amounts of recombinant POWV-ENV proteins (rPOWV-ENV) and irrelevant recombinant protein called gp120 were loaded on a precast 3–12% Bis-Tris NuPAGE gels (Life Technologies, USA) using the Odyssey protein Molecular Weight Marker (LI-COR Biosciences, USA) as a ladder. To detect the loaded recombinant proteins, the immune sera from POWV-SEV vaccinated mice at day 21 were diluted 1:50 and placed overnight at 4°C. The presence of murine POWV-envelope IgG was identified using goat anti-mouse IRDye680CW (LI-COR Biosciences, USA). After washing, the membrane was imaged on the Odyssey infrared imager (LI-COR Biosciences, USA).

For the indirect immunofluorescence assay (IFA), Vero CCL-81 cells were grown in 4-chamber slides (Thermo Fisher, USA), seeded and left to rest for 24 hours. Then, the cells were transfected with 5μg of pMV101 vector only plasmids or POWV-SEV. Forty-eight hours post transfection, cells were fixed for 20 minutes using 4% paraformaldehyde (Sigma-Aldrich, St Louis, USA), then washed in PBST. Cells were incubated with with anti-Flavivirus group antigen antibody (EMD Millipore; Clone D1-4G2-4-15, USA) diluted 1:500 or POWV-SEV vaccinated murine sera (day 21) at 1:50 overnight at 4 °C. The slides were washed as described above and subsequently incubated with goat anti-mouse IgG conjugated with AF488 (Thermo Fisher, USA) for 1 hour at 1:4000. Coverslips were mounted using Fluoroshield Mounting Medium containing 4′,6-diamidino-2-phenylindole (DAPI) (Abcam, USA) and then imaged on a fluorescence microscope (LSM710; Carl Zeiss, USA) as described before [15].

### Animals and DNA immunizations with electroporation

For vaccination, female C57BL/6 mice, 6 to 8 weeks old (The Jackson Laboratory) were housed in a light-cycled, humidity- and temperature-controlled animal facility according to the guidelines from the NIH and the Wistar Institute Institutional Animal Care and Use Committee (IACUC). Murine experiments performed for this study were approved by the Wistar IACUC#201313. Twenty-five μg of pMV101 empty vector or POWV-SEV plasmids were formulated in sterile water, per immunization, and a dose was injected into the anterior tibialis (TA) muscle as previously described [25, 29]. The injection site was immediately supplied with CELLECTRA adaptive constant current enhanced electroporation (EP) delivery device (Inovio Pharmaceuticals, USA), where two 0.1 Amps of triangulated square-wave pulses were delivered per insertion [36]. Each vaccinated group received one or two immunizations of pMV101 or POWV-SEV at two-week intervals. Mice were sacrificed one week after the second immunization at day 21. To collect blood, submandibular puncture was performed prior to each immunization and euthanasia, where mice were anesthetized with 2–5% isoflurane (Phoenix, USA) during procedures.

### Splenocyte isolation and IFN-γ ELISpot assay

Spleens of vaccinated mice were harvested and made into single-cell suspensions using the Stomacher device (Seward, UK). Splenocytes from each mouse were strained into individual 50ml conical tubes (Thermo Fisher, USA) using a 40μm cell strainer (ThermoFisher). The crude splenocytes were lysed for red blood cells for 5 minutes using Ammonium-Chloride-Potassium (ACK) lysis buffer (Quality Biologicals, USA). After washing with PBS, the splenocytes were resuspended in R10 media and the cells were counted using Countess II (Invitrogen). Mouse IFN-γ ELISpot PLUS assay (Mabtech, USA) was performed as per the manufacturer’s instructions.

Briefly, 2x10^5^ splenocytes from the POWV-SEV or pMV101 control immunized mice (*n = 5*) were incubated for 18 hours at 37°C in 5% CO_2_ in three different conditions; along with the splenocytes, the negative control wells contained R10 media alone, the positive control wells contained R10 media with Cell Activation Cocktail (BioLegend, USA) containing pre-mixed phorbol 12-myristate-13-acetate (PMA) and ionomycin, or the POWV envelope-stimulated wells contained POWV envelope linear peptide pools (1 μg/ml). POWV envelope linear pools consist of linearly pooled 20 individual peptides that are 15-mers overlapping by 9 amino acids spanning the length of the POWV envelope protein. After the 18-hour incubation, plates were washed 5 times with PBS. 5-bromo-4-chloro-3-indolyl-phosphate/nitro blue tetrazolium (BCIP/NBT) color development substrate was used to develop blue spots on the wells. Spot forming units (SFU) were quantified by an automated ELISpot reader (CTL Limited, USA). The average number of SFU from the R10 media alone wells was subtracted from each stimulated well, and the data was adjusted to denote SFU per 10^6^ splenocytes [15, 37].

### Indirect enzyme-linked immunosorbent assay (ELISA)

Antigen-specific antibodies were detected using indirect ELISA. MaxiSorp high-binding 96-well ELISA plates (ThermoFisher, USA) were coated with 1μg/mL of recombinant rPOWV envelope protein in PBS at 4°C overnight. Plates were washed 5 times with PBS buffer solution containing 0.01% Tween-20 (PBST) (ThermoFisher, USA), then blocked using 10% FBS in PBS at 37°C for 1 hour. In a separate plate, POWV-SEV-vaccinated murine serum samples (*n = 4*) were prepared and diluted in a half-log fashion in ELISA dilution buffer (1% FBS in PBS), of which 100μl is to be added to each well of the 96-well ELISA plates coated with the recombinant rPOWV envelop protein. Similarly, POWV convalescent human sera were prepared and diluted in a half-log fashion in ELISA dilution buffer (1% FBS in PBS), of which 100μl is to be added to each well of the 96-well ELISA plates coated with the recombinant rPOWV envelop protein. The primary antibodies were then added, incubated at room temperature for 2 hours and subsequently washed with PBST for 5 times. For the secondary antibodies, Goat anti-mouse IgG conjugated with HRP (Sigma-Aldrich, USA) at 1:6000 dilution was used for murine sera, and goat anti-human IgG-HRP (Bethyl) was used for convalescent human sera, which were incubated at 37°C for 1 hour. After washing 5 times with PBST following incubation with secondary antibodies, 100μl of 3,3’5,5’-Tetramethylbenzidine (TMB) Substrate (Sigma-Aldrich, USA) mixed with sterile water was added for 10 minutes before stopping the reaction using 50μl of 2M H_2_SO_4_. The optical density was then measured at 450 nm by Biotek ELISA plate reader (Biotek). For the avidity ELISA to detect the binding strength of POWV immune sera to recombinant envelope protein (rPOWV), 4M of urea (Thermo Fisher, USA) was added for 5 minutes and incubated at room temperature prior to the incubation with secondary antibodies. The antibody endpoint titer was defined as the highest dilution of a serum sample with OD values of pMV101 empty vector injected mice. An endpoint titer of 1 was denoted for samples with a titer <50 as described [38].

Cross-reactivity against various flavivirus antigens was evaluted using indirect ELISA. MaxiSorp high-binding 96-well ELISA plates (ThermoFisher, USA) were coated with 1μg/mL of recombinant rPOWV, rZIKV, rWNV, rDENV1, rDENV2, rDENV3, and rDENV4 envelope proteins in PBS at 4°C overnight. Plates were washed 5 times with PBS buffer solution containing 0.01% Tween-20 (PBST) (ThermoFisher, USA), then blocked using 10% FBS in PBS at 37°C for 1 hour. In a separate plate, POWV-SEV-vaccinated murine serum samples (*n = 4*) were prepared and diluted 1:50 in ELISA dilution buffer (1% FBS in PBS). One hundred microliter (μl) is added to each well of the 96-well ELISA plates coated with the various flavivirus envelop proteins. After the murine serum primary antibodies were added, it incubated at room temperature for 2 hours and subsequently washed with PBST for 5 times. For the secondary antibodies, Goat anti-mouse IgG conjugated with HRP (Sigma-Aldrich, USA) were used at 1:6000 dilution and subsequently treated with TMB substrate and stop solution. The optical density was measured at 450nm Biotek ELISA plate reader (Biotek).

### Flow cytometry and intracellular cytokine staining assay

2x10^6^ single-cell suspended mouse splenocytes were added per well to the U-bottom 96-well plate (ThermoFisher). Cells were stimulated for 5 hours at 37°C in 5% CO2, either in the presence of media alone (negative control), media with Cell Activation Cocktail (BioLegend) containing pre-mixed PMA and ionomycin (positive control), or media with POWV-envelope peptides (1μg/ml) spanning the length of the entire protein, in the presence of GolgiPlug and GolgiStop (BD Biosciences, USA). Upon completed stimulation, the cells are washed with FACS buffer (PBS containing 0.1% sodium azide and 1% FBS). Cells were then stained for the surface proteins using fluorochrome-conjugated antibodies as per the manufacturer’s instructions (BD Biosciences, USA). The cells were washed again with FACS buffer, then fixed and permeabilized using BD Cytofix/Cytoperm (BD Biosciences, USA) as per the manufacturer’s protocol before the intracellular cytokines were stained using fluorochrome-conjugated antibodies (BD Biosciences, USA). The following antibodies were used for surface staining: LIVE/DEAD Fixable Violet Dead Cell stain kit (Invitrogen); CD19 (V450; clone 1D3; BD Biosciences, USA); CD4 (FITC; clone RM4-5; eBioscience, USA); CD8α (APC-Cy7; clone 53–6.7; BD Biosciences, USA); CD44 (A700; clone IM7; BioLegend, USA). For intracellular staining the following antibodies were used: IFN-γ (APC; clone XMG1.2; Biolegend, USA); TNF-α (PE; clone MP6-XT22; eBioscience, USA); CD3ε (PerCP/Cy5.5; clone 145-2C11; Biolegend, USA); IL-2 (PeCy7; clone JES6-SH4; eBioscience, USA). The LSRII flow cytometer was outfitted with the following lasers and bandpass filters: (i) violet (405 nm)-450/50, 525/50, 560/40, 585/42, 605/ 40, 660/40, 705/70, 780/60; (ii) blue (488 nm)-530/30, 695/40; (iii) green (532 nm)-575/25, 610/20, 660/20, 710/50, 780/60; and (iv) red (640nm)-670/30, 710/50, 780/60. All the data were collected using an LSRII flow cytometer (BD Biosciences, USA) and analyzed using FlowJo software (Tree Star, USA) and SPICE v5. Boolean gating was performed using FlowJo software to examine the polyfunctionality of the T cells from vaccinated animals [15].

### POWV viral challenge experiments in C57BL/6 mice

Fourteen C57BL/6 mice were received at University of Texas Medical Branch (UTMB) from The Wistar Institute, PA USA and were provided a one-week acclimation period at UTMB. Half of the mice were designated as having received POWV-SEV immunization (*n = 7*) and half were designated as pMV101 vector immunized (*n = 7*) which served as vaccine control. Baseline clinical observations, body weight measurements, and blood samples were collected prior to POWV challenge. POWV virus with a titer of 4.3 x 10^6^ FFU/mL was diluted with PBS to a final concentration of 3.3 x 10^5^ FFU/mL. Each mouse received a single foot pad injection of 30 μL in the right hind foot using a 26g ½ inch needle for a challenge of 10^4^ FFU dosage as described previously [30]. Following challenge dose, body weights were measured once daily and detailed clinical observations were conducted once daily from days 0–4 and 9–14 and twice daily from days 4–8. Blood samples were collected via the retro-orbital sinus during the experiment and via cardiac puncture at end point or at the end of the study at day 14 dpi. Mice during POWV challenge and retro-orbital blood collection were anesthetized by isoflurane inhalation. Euthanasia was performed by CO_2_ inhalation followed by cervical dislocation. The brain, spleen, liver, skin at injection site, and draining lymph node (right popliteal) were collected from all the mice. Brain, blood, spleen, lymph node and liver samples were stored in Trizol (Fisher, USA) for RNA extraction [30].

### RNA extraction and quantitative Real-Time polymerase chain reaction for viral detection

RNA was extracted from the blood and tissue samples with RNeasy Mini Kits (Qiagen, USA) and quantified on a FS-11x spectrophotometer (DeNovix, USA). Presence of POWV RNA was evaluated by quantitative real-time polymerase chain reaction (qRT-PCR). In accordance with Bio-Rad iTaq Universal SYBR Green One-Step kit instructions for a 20μL reaction, the kit components were combined with POWV-NS5 forward and reverse primers (Integrated DNA Technologies, USA) and up to 1 μg RNA before running qRT-PCR on a CFX96 Touch Real-Time PCR system (Bio-Rad, USA) Viral load quantification was done using a standard curve. RNA was extracted from a sample of known infectivity (1.51 x 10^7^ FFU/mL) and serially diluted prior to running qRT-PCR using the same materials and procedures that were utilized for experimental samples. A linear equation was generated with the resulting threshold cycle (C_T_) values and corresponding log of the viral concentration. The efficiency was 99% (r^2^ value of 0.989). Each qRT-PCR plate which was run included negative control samples containing no sample RNA and multiple dilutions of positive control samples. All the methods used for RNA extraction, viral RNA detection, and standard curve generation have been previously published [30, 39].

### Statistical analysis

Statistical analysis was performed using GraphPad Prism7 (GraphPad software, Inc. USA). All the data were expressed as mean ± standard deviation (SD). Differences between pMV101 and vaccine groups in ELISpot, ELISA, and qPCR were analyzed by a two-tailed unpaired t-test and a Mann–Whitney U-test. The significance of a protective effect conferred by vaccine over POWV-SEV was analyzed by a Log-rank test. Statistical differences between vaccine compared to the pMV101 group were performed to levels of *p < 0.05, **p < 0.01, and ***p < 0.001.
